# Trypanosome SL-RNA detection in blood and cerebrospinal fluid to demonstrate active gambiense human African trypanosomiasis infection

**DOI:** 10.1371/journal.pntd.0009739

**Published:** 2021-09-17

**Authors:** Ipos Ngay Lukusa, Nick Van Reet, Dieudonné Mumba Ngoyi, Erick Mwamba Miaka, Justin Masumu, Pati Patient Pyana, Wilfried Mutombo, Digas Ngolo, Vincent Kobo, Felix Akwaso, Médard Ilunga, Lewis Kaninda, Sylvain Mutanda, Dieudonné Mpoyi Muamba, Olaf Valverde Mordt, Antoine Tarral, Sandra Rembry, Philippe Büscher, Veerle Lejon

**Affiliations:** 1 Department of Parasitology, Institut National de Recherche Biomédicale, Kinshasa, Democratic Republic of the Congo; 2 Department of Biomedical Sciences, Institute of Tropical Medecine, Antwerp, Belgium; 3 Programme National de Lutte contre la Trypanosomiase Humaine Africaine (PNLTHA), Ministry of Health, Kinshasa, Democratic Republic of the Congo; 4 Drugs for Neglected Diseases initiative, Geneva, Switzerland; 5 Mixed Research Unit 177 Intertryp, Institut de Recherche pour le Développement, Centre de Coopération Internationale en Recherche Agronomique pour le Développement, University of Montpellier, Montpellier, France; ITM, BELGIUM

## Abstract

**Background:**

Spliced Leader (SL) trypanosome RNA is detectable only in the presence of live trypanosomes, is abundant and the *Trypanozoon* subgenus has a unique sequence. As previously shown in blood from Guinean human African trypanosomiasis (HAT) patients, SL-RNA is an accurate target for diagnosis. Detection of SL-RNA in the cerebrospinal fluid (CSF) has never been attempted. In a large group of Congolese gambiense HAT patients, the present study aims i) to confirm the sensitivity of SL-RNA detection in the blood and; ii) to assess the diagnostic performance of SL-RNA compared to trypanosome detection in CSF.

**Methodology/Principal findings:**

Blood and CSF from 97 confirmed gambiense HAT patients from the Democratic Republic of Congo were collected using PAXgene blood RNA Tubes. Before RNA extraction, specimens were supplemented with internal extraction control RNA to monitor the extraction, which was performed with a PAXgene Blood RNA Kit. SL-RNA qPCR was carried out with and without reverse transcriptase to monitor DNA contamination. In blood, 92/97 (94.8%) HAT patients tested SL-RNA positive, which was significantly more than combined trypanosome detection in lymph and blood (78/97 positive, 80.4%, *p* = 0.001). Of 96 CSF RNA specimens, 65 (67.7%) were SL-RNA positive, but there was no significant difference between sensitivity of SL-RNA and trypanosome detection in CSF. The contribution of DNA to the Cq values was negligible. In CSF with normal cell counts, a fraction of SL-RNA might have been lost during extraction as indicated by higher internal extraction control Cq values.

**Conclusions/Significance:**

Detection of SL-RNA in blood and CSF allows sensitive demonstration of active gambiense HAT infection, even if trypanosomes remain undetectable in blood or lymph. As this condition often occurs in treatment failures, SL-RNA detection in blood and CSF for early detection of relapses after treatment deserves further investigation.

**Trial registration:**

This study was an integral part of the diagnostic trial "New Diagnostic Tools for Elimination of Sleeping Sickness and Clinical Trials: Early tests of Cure" (DiTECT-HAT-WP4, ClinicalTrials.gov Identifier: NCT03112655).

## Author summary

Human African trypanosomiasis is a parasitic infection occurring in sub-Saharan Africa, which is fatal if left untreated. Diagnosis relies on demonstration of trypanosomes, which may occur at such low concentrations that they remain microscopically undetectable. Nucleic acid detection offers an alternative, in particular RNA, which is unstable and a better marker for live organisms than DNA. Trypanosomal SL-RNA detection in blood by reverse transcriptase quantitative PCR has hitherto only been tested twice. Although in cerebrospinal fluid, trypanosome presence indicates brain infection, SL-RNA detection has never been attempted. We evaluated sensitivity of SL-RNA detection in blood and cerebrospinal fluid. For each specimen, 2 controls were included: presence of genomic DNA contamination and efficacy of RNA extraction. Sensitivity of SL-RNA detection in blood was higher than of combined blood and lymph microscopy. In cerebrospinal fluid, SL-RNA and trypanosome detection had similar sensitivity. In a few specimens, traces of DNA were amplified. In some cerebrospinal fluids, some RNA was lost during extraction. Performing both internal controls is crucial, to ensure that negative SL-RNA cerebrospinal fluid findings are not due to a failed extraction and, in particular when testing treated patients, to differentiate live parasite RNA from reminiscent DNA.

## Introduction

Human African trypanosomiasis (HAT) or sleeping sickness is a neglected tropical disease caused by *Trypanosoma brucei* (*T*.*b*.) *gambiense* in Central and West Africa and by *T*.*b*. *rhodesiense* in East Africa. In humans, presence of the trypanosome in the blood and lymph only, corresponds to the first stage of the disease, whereas the second stage is characterized by trypanosome invasion of the central nervous system.

The diagnosis of HAT is primarily based on the detection of the parasite in the blood, lymph or cerebrospinal fluid (CSF) [1]. Performance of diagnostic tests is influenced, for *T*.*b*. *gambiense* HAT, by the low and fluctuating trypanosomal load in these body fluids, intrinsic to the parasite and its interaction with the host’s immune response. The resulting low sensitivity of microscopic diagnosis, implies a risk of failure to detect the parasite although a patient is actually infected [2].

PCR targeting parasite DNA presents an alternative for the diagnosis of HAT [3]. However, DNA detection does not fully differentiate active infection, when the parasite is present, from a cleared infection. In some patients, long-term persistence of DNA has been observed, even after successful treatment [4,5].

The detection of trypanosomal RNA, which is unstable and produced only by live parasites, demonstrates an active infection in the host and thus enables to establish a definite diagnosis. Spliced Leader (SL) trypanosome RNA can be used for diagnostic purposes, as it is abundant in trypanosomes and the *Trypanozoon* subgenus has a unique sequence. SL-RNA is involved in the maturation of messenger RNA to which it is attached [6]. The diagnostic sensitivity of SL-RNA detection in blood of sleeping sickness patients from Guinea with trypanosomes detected in blood and/or lymph reached 91.7–96.7%, while in the same studies specificities were 95.5 and 100% [5,6]. Also for post-treatment follow-up of patients with *T*.*b*. *gambiense*, the specificity of SL-RNA detection in blood was higher than that of DNA detection by PCR [5]. To date, SL-RNA detection has never been applied on the blood of gambiense HAT patients from other countries. Also, the diagnostic performance of SL-RNA demonstration in CSF of HAT patients remains unknown.

Although presence of trypanosomes in CSF has traditionally been a marker for 2^nd^ stage HAT, stage determination loses importance as new stage-independent treatments for sleeping sickness emerge [7–9]. Nevertheless, parasitological examination of CSF remains relevant. A fraction of gambiense HAT patients can only be confirmed by trypanosome detection in CSF, despite using the most sensitive techniques on blood [10]. Also for detection of relapses, CSF examinations are usually more sensitive than parasitology on blood [11,12]. The present study therefore aims i) to confirm the sensitivity of SL-RNA detection in the blood of a large group of gambiense HAT patients from the Democratic Republic of Congo (DRC) and; ii) to assess the diagnostic performance of SL-RNA detection in CSF of those patients.

## Methods

### Ethics statement

This study was an integral part of the diagnostic trial "New Diagnostic Tools for Elimination of Sleeping Sickness and Clinical Trials: Early tests of Cure" (DiTECT-HAT-WP4, ClinicalTrials.gov Identifier: NCT03112655). The DiTECT-HAT-WP4 study has received approval from the “Comité Consultatif de Déontologie et d’Ethique” (CCDE) of the French National Institute for Sustainable Development Research (IRD, June 2016 session), from the institutional Review Board of the Institute of Tropical Medicine (reference 1096/16), and from the Ethics Committees of the Ministry of Health, DRC through the Ngaliema Clinic of Kinshasa (September 2016 session) and the School of Public Health of the University of Kinshasa (reference ESP/CE/136/2020). It was carried out in conjunction with a prospective therapeutic trial conducted by DNDi (DNDi-OXA-02-HAT, registered as NCT03087955) on the new drug Acoziborole for HAT that was approved by the CERSAC, Ethics Committees of the Ministry of Health, DRC through the Ngaliema Clinic of Kinshasa and the CNES (Comité National d’Ethique de la Santé), DRC.

Participants first gave their informed consent to participate in the therapeutic trial. A separate written informed consent was obtained next before their inclusion in the DiTECT-HAT-WP4 study. For minors participating in the DiTECT-HAT-WP4 study, an additional written consent was obtained from their legal representative.

### Study sites

The DiTECT-HAT-WP4 study was conducted in Democratic Republic of the Congo from February 2017 until March 2019. Patients were recruited in five reference district hospitals that were conducting the DNDi-OXA-02-HAT therapeutic trial under the supervision of the PNLTHA, located in Dipumba and Katanda in the province of Kasaï Oriental, Ngandajika in the province of Lomami, Masi Manimba in the province of Kwilu and in Kinshasa.

### Patients

Patients included in the DITECT-HAT-WP4 study were enrolled first in the DNDi-OXA-02-HAT study. The inclusion criteria for DiTECT-HAT-WP4 corresponded to those applied in the DNDi-OXA-02-HAT study: confirmed HAT with *T*.*b*. *gambiense* parasites in blood, lymph and/or CSF; age ≥15 years; give and sign the study consent form; Karnofsky index > 50; able to take tablets orally; have a permanent address or being retrievable by other people; able to comply to the follow-up visits schedule and to other constraints of the study and accept to be hospitalized for acoziborole treatment and study procedures. As all HAT patients participating in DiTECT-HAT-WP4 were first enrolled in the DNDi-OXA-02 study, they were treated with acoziborole, independent of their disease stage.

### Diagnosis

Patients included in this study were diagnosed either in the village by mobile teams (active screening), or at the reference district hospital (passive screening). Screening consisted of identifying characteristic clinical signs, in particular swollen cervical lymph nodes, and serological tests (CATT/*T*.*b*. *gambiense* test or a rapid diagnostic test for gambiense HAT). Individuals who were positive in a serological test underwent parasitological examination. For persons with swollen cervical lymph nodes, a lymph aspirate was examined for trypanosomes by microscopy. For persons positive in a serological test but negative in lymph or without swollen lymph nodes, blood was examined for trypanosome presence by mini anion-exchange centrifugation technique on buffy coat (mAECT-BC)[13]. If mAECT-BC was unfeasible, blood was examined by mAECT or the haematocrit centrifugation technique. All HAT patients detected by active screening were reconfirmed at the reference district hospital before inclusion. After parasitological confirmation, or if there was a strong clinical and serological suspicion, a lumbar puncture was performed. The CSF was examined for white blood cell (WBC) number. Trypanosomes were searched for during the cell counting procedure, and, if no trypanosomes had been observed, by the modified single centrifugation technique of the CSF [14]. The patient was classified as early or intermediate stage with absence of trypanosomes in the CSF and a WBC count in the CSF of respectively ≤5 or 6–20 WBC/μl. The late stage of the disease was defined by the presence of trypanosomes in the CSF or >20 WBC/μl. As a quality control, video recordings were made to certify presence of viable trypanosomes in lymph, blood and/or CSF.

From each HAT patient, 2.5 ml of blood were collected in a PAXgene Blood RNA Tube (PreAnalytiX, www.PreAnalytiX.com) according to the instructions of the manufacturer. After the collection of CSF for direct examination, the PAXgene Blood Tube was opened and 2.5 ml of CSF were collected by holding the tube under the lumbar puncture needle, after which the tube was closed. Both blood and CSF samples collected in PAXgene Blood RNA Tubes were inverted at least 10 times, and decanted immediately into 10 ml cryotubes. They were stored at -20°C for maximum 2 months before transport in liquid nitrogen to the HAT National Reference Laboratory at the Institut National de Recherche Biomédicale (INRB) in Kinshasa, where they were stored at -80°C until RNA extraction.

### RNA extraction

RNA extraction on blood and CSF samples collected in PAXgene Blood RNA Tubes was performed using a PAXgene Blood RNA Kit IVD following the guidelines of the manufacturer (www.PreAnalytiX.com, PAXgene Blood RNA Kit Handbook 04/2008). The samples were thawed by placing them at ambient temperature for 4 hours to ensure full lysis before centrifugation and start of the extraction process. The pellet was lyzed and supplemented after centrifugation with 4 μl RNA internal extraction control (IEC) according to manufacturer’s recommendations (Primer design Ltd, York House, School Lane, Chandlers Ford, UK SO53 4DG). Briefly, the resuspended pellet was proteinase K treated, and residual cell debris removed through PAXgene shredder spin column centrifugation. Ethanol was added to the flow-through fraction and the lysate applied to a PAXgene RNA spin column centrifugation. The bound RNA was treated with DNase I and after several wash steps, RNA was eluted in 80 μl and stored at -80°C. RNA extracted from human blood spiked with *T*.*b*. *gambiense* LiTat 1.3 served as positive control.

### SL-RNA quantitative PCR

SL-RNA was amplified using SensiFAST SYBR Lo-ROX One-Step Mix (Bioline, United Kingdom) with 400 nM of primers 5′-CAATATAGTACAGAAACTG-3′ (cSL) and 5′-AACTAACGCTATTATTAGAA-3′ (SL-F) (IDT Integrated DNA Technologies) and 4 μl RNA eluate in a final volume of 20 μl. All RNA extracts were tested in duplicate, and with (RT) or without (noRT) addition of reverse transcriptase (RT) (Bioline, United Kingdom). Cycling conditions using AriaMX Real-Time PCR System Machine (Agilent Technologies, USA) were 45°C for 10 minutes, 95°C for 2 minutes, and 40 cycles of 95°C for 2 seconds, 50°C for 10 seconds and 60°C for 5 seconds. The melting curve was recorded between 45°C and 95°C, using increments of 0.5°C for 5 seconds. Each qPCR run included a positive control from the spiked *T*.*b*. *gambiense* RNA extract and a negative control.

An RNA extract was considered positive for SL-RNA if both RT replicates were associated with a melting temperature (Tm) between 65 and 68°C. When detected, the Cq of the noRT reaction was used to calculate the percentage contribution of genomic DNA to the Cq-value of the RT reaction according to the following equation [15]:

$$\%gDNA=\left(\frac{2^{-Cq\;noRT}}{2^{-Cq\;RT}}\right)\;x\;100$$

### Internal extraction control qPCR

The IEC was amplified using qScript XLT One-Step RT-qPCR ToughMix, Low ROX, using 1 μl of IEC primer/probe (Primer design Ltd, UK) and 5 μl of RNA extract in a 20 μl reaction. Cycling conditions using AriaMX Real-Time PCR System Machine (Agilent Technologies, USA) were 50°C for 10 minutes, 95°C for 2 minutes and 40 cycles of 95°C for 2 seconds, 50°C for 10 seconds and 60°C for 5 seconds.

### Data analysis

For proportions, binomial confidence intervals with a 95% confidence level (CI) were calculated. The categorical results of trypanosome and SL-RNA detection in the same patients were compared by the Mc Nemar Chi square test. The Cq values of the IEC of two groups of patients were compared by an unpaired t-test, determining the two-tailed *p* value.

## Results

### Patient characteristics

In total, 97 parasitologically confirmed HAT patients were included in DiTECT-HAT-WP4, 53 in Masi Manimba, 23 in Dipumba, 9 in Katanda, 10 in Ngandajika and 2 in Kinshasa. The median age of the participants was 31 years (interquartile range 24–44). The sex ratio was 50 females for 47 males. The CATT test was positive in 100% of patients on whom it was performed (95/95), the remaining two patients, on whom CATT was not performed, were positive in SD Bioline HAT. The parasitological results of these HAT patients are summarized in Table 1.

**Table 1:** Results of RT-qPCR on blood and CSF from 97 gambiense HAT patients by disease stage, according to demonstration of trypanosomes in blood, lymph and CSF, and to CSF WBC count.

		SL-RNA	
	N	Blood positive	CSF positive
**Early stage (N = 14)**			
Tryp. in lymph and/or blood, no Tryp. in CSF, WBC<5	14	14	0
**Intermediate stage (N = 7)**			
Tryp. in lymph and/or blood, no Tryp. in CSF, WBC 5–20	7	7	1
**Late stage (N = 76)**		**71**	**64/75** *
Tryp. in lymph and/or blood, Tryp. in CSF, WBC <20	3	3	0
Tryp. in lymph and/or blood, Tryp. in CSF, WBC >20	46	45	44/45*
Tryp. in lymph and/or blood, Tryp. in CSF, WBC not done	1	1	1
Tryp. in lymph and/or blood, no Tryp. in CSF, WBC >20	7	7	2
No tryp. in lymph and/or blood, Tryp. in CSF, WBC <20	1	1	0
No tryp. in lymph and/or blood, Tryp. in CSF, WBC >20	18	14	17

N = number of patients; CSF = cerebrospinal fluid; Tryp. = trypanosome

* CSF specimen missing for 1 patient; WBC = CSF white blood cell count/μl

Swollen lymph nodes could be punctured in 53 patients, and lymph was trypanosome positive for 35/53 HAT patients included in the study (66.0%, CI 52.6–77.4%). Next, blood was examined for 68 patients, and trypanosomes were detected in blood for 45 of 68 included HAT patients (66.2%, CI 54.3–76.3%). Lymph or blood were trypanosome positive in 78/97 HAT patients (80.4%, CI 71.1–87.8%). In total 69/97 (71.1%, CI 61.0–79.9%) HAT patients had trypanosomes in CSF, including all 19 patients in whom trypanosome infection could not be demonstrated previously in lymph and blood.

**Fig 1:**
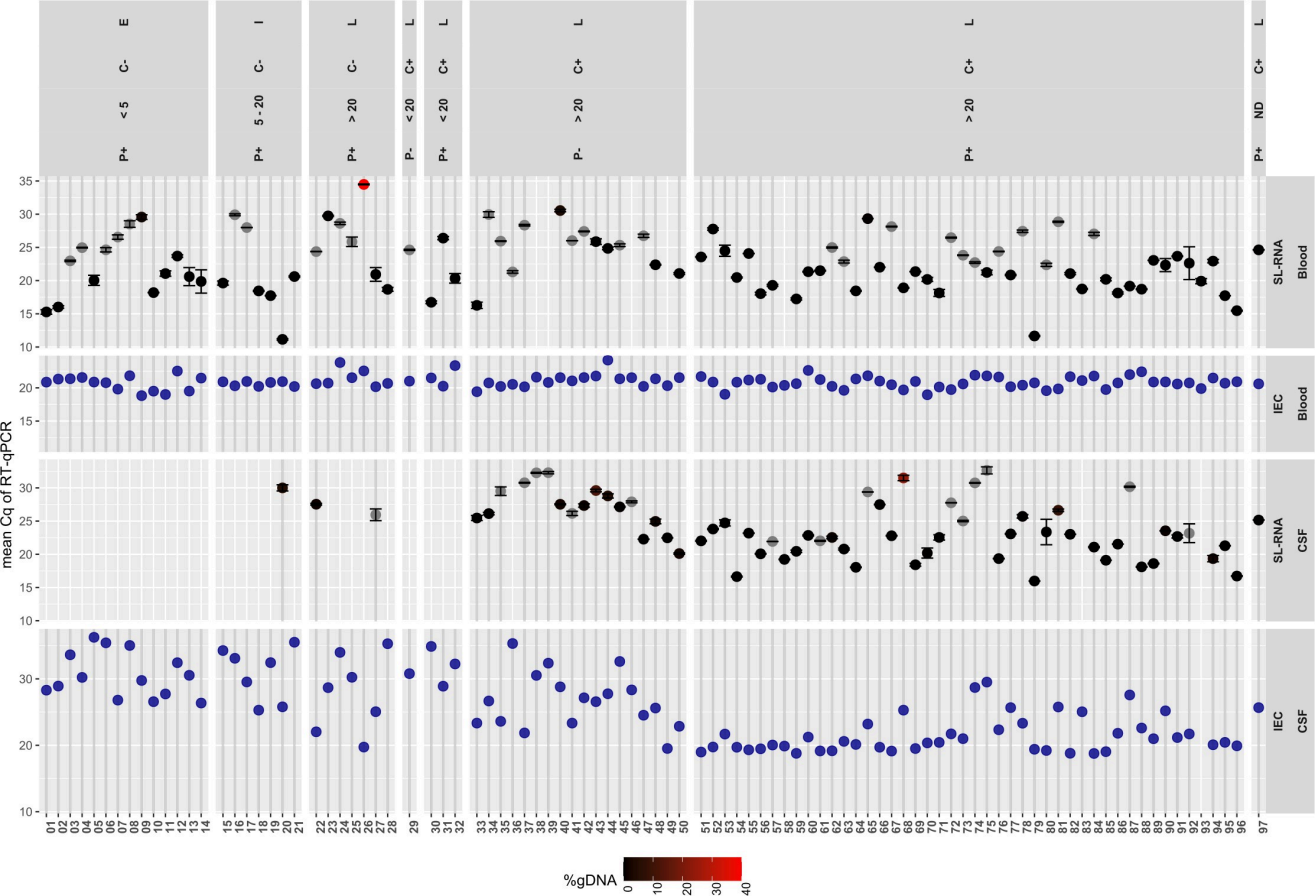
Cq values of RT-qPCR SL-RNA detection in blood and CSF of gambiense HAT patients. The results are combined with RT-qPCR for internal extraction control (IEC), according the disease stage (E early, I Intermediate and L late stage), parasitological test result in blood and lymph (P), parasitological test result in CSF (C), and CSF WBC count/μl. + trypanosome presence;—absence of trypanosomes. ND not determined.

Fourteen HAT patients were classified in early stage (14.4%, CI 8.1–23.0%), 7/97 in intermediate stage (7.2%, CI 3.0–14.3%) and 76/97 in late stage (78.4%, CI 68.8–86.1%). All of them were SL-RNA positive, either in CSF and/or blood. Detailed results are shown in Fig 1.

### RT-qPCR results on blood specimens

The internal extraction control added to each blood specimen during the RNA extraction procedure was detected by RT-qPCR in all 97 extracted blood specimens with a mean Cq value of 20.9 (standard deviation 1.0).

Of 97 confirmed HAT patients, 92 (94.8%, CI 88.2–98.1%) tested SL-RNA positive in blood (Table 1). The contribution of genomic DNA to the RT detection of SL-RNA could not be calculated for 30 reactions since no amplification in noRT replicates was observed (grey symbols in Fig 1), was below 4% in 61 reactions and was 39% in one reaction (black to red gradient in Fig 1).

Four out of 5 SL-RNA negative specimens (patients 38, 39, 46, 58) did not show amplification (no Cq value) and/or had a RT Cq value <40 but a melting temperature far outside the 65–68°C range. One negative specimen (patient 49) showed one positive reaction (Cq 33.6 and melting temperature 66°C) and one negative reaction (no Cq value). In only one of these blood SL-RNA negative patients, trypanosomes had been detected in blood and CSF (patient 58), while in the other 4 (patients 38, 39, 46, 49), trypanosomes had been detected in CSF only. Video recordings reconfirmed microscopic parasite finding for these 5 SL-RNA negatives. In the 68 patients in whom trypanosome presence in blood had been examined, SL-RNA was significantly more positive than trypanosome detection (*p* = 0.0001, 63 SL-RNA versus 45 parasite positives). SL-RNA detection in blood was also significantly more positive than combined lymph and blood parasite detection (*p* = 0.001, 92 SL-RNA versus 78 blood and/or lymph parasite positives in 97 HAT patients).

Of 14 patients in early stage and 7 in intermediate stage, respectively 14 (100%, CI 76.8–100%) and 7 (100%, CI 59.0–100%) were SL-RNA positive. Of 76 patients in late stage, 71 (94.7%, CI 85.2–97.5%) had SL-RNA positive results (Table 1).

### RT-qPCR results on CSF specimens

**Fig 2:**
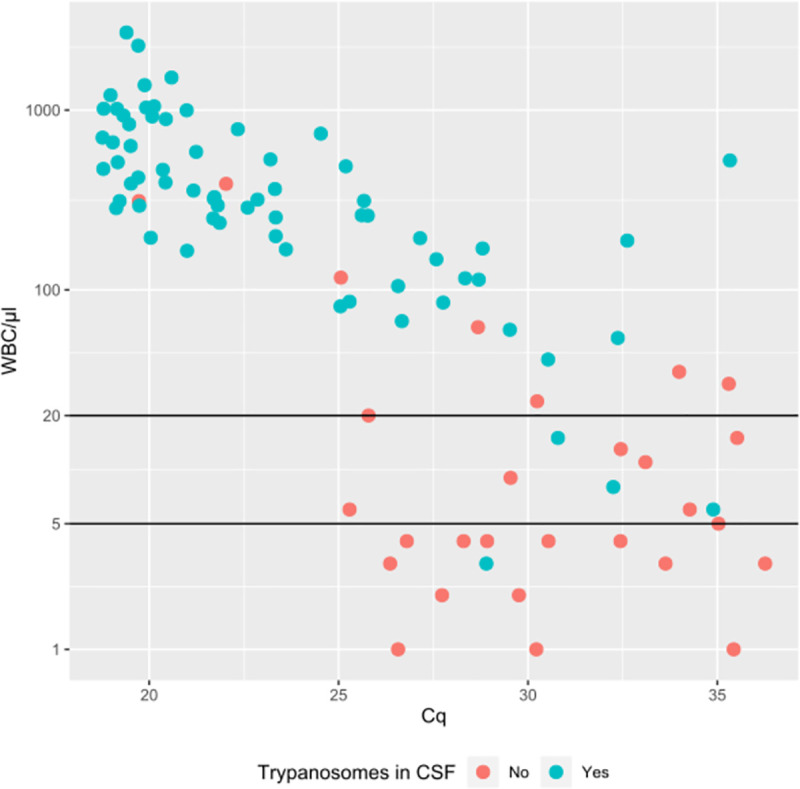
Cq values of the internal extraction control RT-qPCR in CSF in function of the CSF white blood cell count/μl. Blue dots: trypanosomes in CSF. Red dots: no trypanosomes in CSF.

Of 97 patients included, 96 CSF RNA specimens were available (CSF RNA specimen of patient 93 missing). The internal RNA extraction control added to each CSF specimen during the RNA extraction procedure was detected by RT-qPCR in all 96 specimens with a mean Cq corresponding to 25.4 (standard deviation 5.3, Fig 1). As shown in Fig 2, extraction of RNA appeared more efficient (low IEC Cq values) in CSF specimens with high WBC counts, and decreased with the WBC count, as witnessed by increasing IEC Cq values. There was a significant difference (p<0.0001) between the IEC Cq of 30.8 (standard deviation 3.4, n = 25) of CSF with WBC count ≤ 20/μl, and IEC Cq of 23.4 (standard deviation 4.4, n = 70) of CSF with WBC counts of >20/μl, indicating more efficient RNA extraction in the latter (WBC count not available for 1 specimen).

In CSF, 65/96 patients (67.7%, CI 57.8–76.35%) were SL-RNA positive (Table 1). Again, the gDNA contribution to RT SL-RNA detection was absent or small (Fig 1): 1 RNA extract was detected with 24% gDNA and 1 with 12%, 10 RNA extracts contained between 10–1% gDNA, 37 extracts had < 1% gDNA contribution, while in 16 RNA extracts no noRT reaction was observed. For eight SL-RNA negative patients, one RNA replicate tested positive while the other tested negative (patients 6, 12, 17, 23, 26, 31, 32, 83).

Of 14 early stage patients, zero (0.0%, CI 0.0–25.2%) had SL-RNA in CSF, while of 7 patients in intermediate stage, one (14.3%, CI 0.53–53.4%) reacted SL-RNA positive (patient 20). Of 75 CSF specimens from late stage patients, 64 (85.3%, CI 75.4–91.8%) contained SL-RNA. From 7 patients in late stage without trypanosomes in CSF, 2 (28.6%, CI 7.6–64.8%) were CSF SL-RNA positive (patients 22 and 27). From 68 late stage CSF samples containing trypanosomes, 62 (91.2%, CI 81.7–96.2%) were SL-RNA positive. In 6 patients who were SL-RNA negative but reported as modified single centrifugation positive (patients 29–32, 36 and 83), retrospective analysis of the video recordings reconfirmed presence of live trypanosomes in the CSF. There was no significant difference in the number of SL-RNA or trypanosome positives (*p* = 0.5, 65 SL-RNA versus 68 modified single centrifugation positives).

## Discussion

The obtained results confirm the high sensitivity of SL-RNA detection in blood for diagnosis of HAT, which exceeds the sensitivity of parasitological diagnosis in blood and lymph. For the first time, trypanosomal RNA was detected in the CSF of HAT patients, and the technique has similar sensitivity as the single modified centrifugation of CSF for trypanosome detection.

A number of strengths from the present study merit to be highlighted. An important improvement was the integration of an internal extraction control, through the addition of exogenous RNA to the test specimens, in order to verify the efficiency of the RNA extraction process. In particular for CSF, which was processed for the first time, inclusion of this extraction control seems a must when specimens with relatively normal WBC counts (≤20/μl) are processed. A second control step introduced in the protocol was parallel qPCR analysis of the specimen without addition of reverse transcriptase. This enables the calculation of the contribution of genomic DNA to the obtained RNA result, thus guaranteeing that RNA was effectively detected instead of remnant DNA. Moreover, as it has been estimated that half-live of most trypanosomal mRNA is less than 100 minutes [16], the detection of SL-RNA in a specimen thus implies ongoing active *Trypanozoon* infection. This is of particular importance once specimens of treated patients will be analysed, as trypanosomal DNA may circulate long after cure, especially in CSF. Indeed, previous observations suggest presence of trypanosomal DNA in respectively 24.6% and 35.4% of blood and CSF of successfully cured patients, even between 12 and 24 months after HAT treatment [4]. Another strength is the collaboration with a therapeutic clinical trial, which led to the collection of well-characterized and high quality specimens, with a quality control on positive parasitological tests through video recordings. This allowed re-confirmation of parasite positive results *a posteriori*. Similar therapeutic trials will probably not be conducted anymore in the near future as different new oral drugs have emerged to treat all stages of HAT, implying that the systematic lumbar punctures for disease staging will no longer be needed. The treatment regimen with fexinidazole requires a lumbar puncture only if clinical signs suggest severe second stage HAT [9], which is usually accompanied by a cytorachia of more than 100 WBC/μl [17]. In the future, lumbar puncture will probably be limited to clinically suspected HAT cases in whom no trypanosomes were detectable in lymph or blood and that need parasitological confirmation in CSF, including relapses after treatment. The present trial was therefore probably the last opportunity to test the feasibility of RNA extraction and detection in a larger number of gambiense HAT patient CSF specimens.

The present study also has some limitations. First, although we were able to demonstrate the high sensitivity of RNA detection in blood of HAT patients, we could not determine the specificity of the test, as no specimens from non-HAT control individuals had been collected. However, in 2 previous studies using a similar protocol in Guinea, specificities of respectively 96 and 100% were observed in 49 and 32 endemic healthy controls [5,6]. The strong disparity of the Cq values observed for the internal extraction control in CSF, might indicate that the PAXgene collection and extraction protocol, which is efficient for blood, is suboptimal for the purification of RNA from normal non-inflammatory CSF, which, due to the low WBC number, probably has a low total RNA content. Finally, none of the participating HAT patients has been tested for presence of trypanosomes in the skin [18,19]. Although it cannot be excluded that blood parasitemia in some patients was below the detection limit of mAECT-BC, lymph or skin trypanosomes could be at the origin of RNA detected in their blood.

In this study, the sensitivity of SL-RNA detection in the blood for diagnosis of HAT was 95.8% (CI 89.8–98.8%). In the past, the value of this parameter was determined at 92% (CI 78–97%) [6] and also at 96.7% (CI 88.7–99.6%), and 95.1% (CI 86.3–99.0%) [5]. However, in those previous studies, trypanosomes had always been detected in lymph and/or blood of the tested individuals, which was not the case here. In the present study, for 19% of the participants, confirmatory diagnosis of HAT was not obtained in lymph or blood and could only be obtained through the examination of the CSF. A previous study [10], which was also conducted in Kwilu, one of the actual study areas in DR Congo, also observed that 4% of gambiense HAT patients are only confirmed by the detection of trypanosomes in the CSF, despite the use of the most sensitive techniques on the blood. This not only underlines the importance of CSF examination for diagnosis of HAT in cases of high suspicion, but also the ability of SL-RNA detection in blood to pick up such patients. This observation also suggests that SL-RNA detection in blood could be an appropriate candidate for blood diagnosis of relapses after treatment, which often present with trypanosomes in the CSF only [11,12].

Detection of trypanosomes in CSF is a marker for the diagnosis of second stage HAT [1]. However, it is not always easy to detect the parasite in this fluid because it is known to be an unsuitable environment for survival of the trypanosome [20]. SL-RNA detection in CSF also stood out for its performance, with a sensitivity assessed at 95.6% (87.6–99.0%) compared to parasitological tests in CSF. There was no significant difference between SL-RNA detection in CSF and modified single centrifugation. The study found that 3 patients with negative CSF parasitology, including one patient in intermediate stage, had a positive SL-RNA test in CSF. In fact, also PCR has sometimes been positive in CSF where CSF parasitology remained negative, or in early or intermediate stage patients [21,22]. In addition, it is known that stage 1 and intermediate stage patients may be PCR positive in the CSF but still successfully treated with first stage drugs [4,23,24]. Although in such cases, PCR positivity could be explained by presence of trypanosome DNA in the CSF without actual presence of the parasite [4,23], presence of SL-RNA in CSF as demonstrated in the actual study, seems to confirm presence of live trypanosomes and active infection. In experimental models, trypanosomes indeed pass the blood brain barrier and are able to invade the central nervous system shortly after entering the bloodstream, and before meningoencephalitis is established [25]. Caution has therefore already been expressed about the interpretation of positive PCR results in otherwise normal CSF [4,26], and the same might apply to RNA detection.

The high sensitivity of SL-RNA RT-qPCR opens the door for future diagnostic applications. However, as for PCR in the past, SL-RNA is, for the moment, not really suitable for routine diagnosis of HAT under field conditions in rural areas where HAT occurs. It requires demanding and critical logistics, including correct specimen collection on RNA stabilization buffer, and transport of the stabilized RNA soon after collection or frozen from the HAT treatment center to a reference lab for proper RNA extraction, which cannot be expected in routine [3]. In addition, RNA collection materials and RNA extraction remain relatively expensive. Complexity of specimen collection and processing, affordability and need of specific equipment therefore represent the main barriers for implementation of RT-qPCR for diagnosis of HAT. In particular circumstances, RNA detection could however correct the shortcomings of DNA detection, especially when high sensitivity is required such as in latent infections, which do not progress to clinical disease and may remain undiagnosed [27], or when demonstration of active infection is critical and DNA detection is not reliable, such as for diagnosis of relapse after treatment [4,5]. Another potential application could be the *a posteriori* confirmation of HAT in serological suspects who undergo treatment without parasitological examination, as could be the case in future “test and treat” algorithms [28].

Future research should confirm specificity of RNA detection and confirm its sensitivity in CSF. The performance of detection of SL-RNA as a marker for the diagnosis of relapses during post-treatment follow-up of patients with *T*.*b*. gambiense should be explored further. In particular the high sensitivity of RNA detection in blood could open the door for less invasive diagnosis of relapses, avoiding lumbar puncture. Furthermore, as recently new RNA detection tests have emerged which have only been tested experimentally, including 7SL-derived small RNA [29], future research should also include evaluation of the diagnostic performance of detection of new potentially more sensitive RNA targets on blood and CSF specimens from HAT patients.

In conclusion, with the present study, we confirmed the elevated sensitivity of SL-RNA detection in the blood of HAT patients and demonstrated the diagnostic performance of SL-RNA detection in CSF for the first time, which is a first step towards new applications of RNA detection.
